# From Earth to Mars: a perspective on exploiting biomineralization for Martian construction

**DOI:** 10.3389/fmicb.2025.1645014

**Published:** 2025-12-02

**Authors:** Shiva Khoshtinat, Jared Long-Fox, Seyed Mohammad Javad Hosseini

**Affiliations:** 1 Department of Chemistry, Materials and Chemical Engineering “Giulio Natta”, Politecnico di Milano, Milan, Italy; 2 Department of Physics, University of Central Florida, Orlando, FL, United States; 3 School of Environment and Safety Engineering, Jiangsu University, Zhenjiang, China

**Keywords:** Mars, construction, biomineralization, *in situ* resource utilization, robotics, additive manufacturing

## Abstract

The future of Mars colonization hinges on the ability to construct durable infrastructure using locally available resources. Given the high cost and logistical complexity of transporting construction materials to Mars, the development of autonomous *in situ* resource utilization (ISRU) technologies is imperative. This perspective article explores the potential of biomineralization as a low-energy, sustainable alternative to conventional construction methods, such as Portland cement and thermal sintering approaches proposed for lunar applications, which are often energy-intensive and constrained by material specificity. Following an assessment of the chemical composition of Martian regolith, its suitability as a substrate for various biomineralization pathways, particularly those aligned with ISRU constraints, is evaluated. Special emphasis is placed on identifying biological pathways that are not only metabolically compatible with Martian geochemistry but can also function as a co-culture, mutually supporting each other’s survival and activity under Martian environmental stresses. The most promising microbial consortia for biocementation are proposed for future extraterrestrial construction applications. The integration of robotics and automation in biocementation-based additive manufacturing using Martian regolith as a construction feedstock is discussed. Advanced robotic systems equipped with multi-axis extrusion nozzles, sensor suites, and real-time flow control are proposed for the construction of structurally resilient geometries on Mars. As a flexible, scalable, and ISRU-compatible technology, biocementation holds promise not only for infrastructure construction but also for integrated resource cycles, producing oxygen and ammonia as byproducts. Biocementation-based ISRU construction represents a synergistic pathway toward sustainable human presence on Mars, enabling robotic fabrication of critical infrastructure from locally available materials.

## 1 Introduction

Human colonization of other planets has long been envisioned, but its realization relies on the ability to construct safe and sustainable infrastructure off-Earth. Mars is thought to have a relatively similar geological history to Earth and hence has long served as a target of fascination and robotic exploration missions and is a leading candidate for future human settlement. However, long-term human and robotic presence on Mars remains highly challenging due to extreme environmental conditions, such as a thin atmosphere that freezes and thaws throughout the Martian day, extreme radiation levels, and wide temperature fluctuations, as well as significant power and time constraints for mission operations. Global space agencies and organizations, such as the United States National Aeronautics and Space Administration (NASA), aim to develop human habitats on Mars (NASA, 2022). However, substantial technological and scientific advances are required to ensure safe, efficient, and cost-effective exploration and infrastructure development. A critical challenge is the sourcing of construction materials, as transporting them from Earth is prohibitively expensive and constrained by launch costs, limited payload mass, and payload volume constraints (Naser, 2019; Wang et al., 2022). Therefore, *in situ* resource utilization (ISRU) is critical for human and robotic missions’ sustainability beyond Earth (Sanders et al., 2022). Martian regolith is analogous to terrestrial soil and is abundant in oxygen and extractable metals (Schreiner et al., 2015), offering a viable resource for local construction, manufacturing, and propellant production (Farries et al., 2021). It can be used as a bulk material for infrastructure such as landing pads, berms, habitats, and roadways, significantly reducing reliance on Earth-based supply chains for the development of Martian infrastructure.

Given the high risk and cost associated with human spaceflight and off-Earth construction, autonomous or remotely operated robotic systems offer advantages by enhancing safety and construction consistency. Robotic systems equipped with imaging and geotechnical instruments, such as vane shears, cone penetrometers, and bearing plates, can be used to evaluate potential construction sites, additively manufactured parts, and infrastructure element mechanical properties (e.g., compressive strength and shear strength) before, during, and after construction. Such tools have been successfully deployed on lunar and Martian missions (Zent et al., 2009), demonstrating their effectiveness in assessing environmental and regolith properties. However, the mechanical behavior of planetary regolith differs significantly from terrestrial soils (Long-Fox and Britt, 2023; Long-Fox et al., 2023; Dotson et al., 2024; Lucas et al., 2024), necessitating careful adaptation and calibration of measurement techniques. Employing robotic geotechnical tools and imaging systems improves the accuracy, repeatability, and reliability of site assessments, ultimately reducing time, energy, and cost in extraterrestrial construction.

Cement is the most predominant construction material on Earth (Hosseini et al., 2024b). In recent years, biomineralization, a process in which microorganisms facilitate mineral formation under ambient conditions with low energy input (Zhu et al., 2024), has garnered considerable attention as a sustainable cement alternative. The biomineralization process typically begins when microbes adhere to a substrate containing the requisite ions for mineralization (Lowenstam et al., 1989). A key element in microbial–mineral interactions is the secretion of extracellular polymeric substances (EPS), a matrix of polysaccharides, proteins, and sometimes DNA fragments that enhances surface adhesion (Dupraz and Visscher, 2005). Within the EPS, microorganisms concentrate essential ions, creating a microenvironment that favors mineral nucleation and growth by altering local environmental parameters such as pH, redox conditions, or ion saturation (Lowenstam et al., 1989). Nucleation begins when ions aggregate to form a stable crystal “seed” on organic or inorganic surfaces. Once nucleated, the mineral crystals grow under biological control, often influenced by organic molecules that bind to specific crystal faces and regulate morphology (Mann, 2001). The final stage often involves the hardening, aging, or remodeling of the mineralized structure, including transitions from amorphous to crystalline phases (Mann, 2001).

This manuscript explores the feasibility of employing biomineralization for Martian construction. First, the chemical composition of Martian regolith is analyzed since the composition of the regolith determines the feasibility and best approach to biomineralization. Then, various biomineralization pathways are presented with an emphasis on those that can exploit native Martian minerals as potential substrates. This work then further explores how Mars’ extreme environmental conditions may constrain biomineralization processes and influence their effectiveness. The authors assess the viability of biomineralization for Martian construction, highlighting the most promising pathways, potential integration with ISRU, and the prospects for automation and remote operation. Finally, key challenges and current research gaps are discussed.

## 2 Martian resources and environmental challenges for biomineralization

### 2.1 Martian soil composition

Martian regolith has only been analyzed through data collected by surface robotic or orbital missions. Despite regional variations, its chemical composition shares similarities with that of Portland cement, particularly in key oxides. As shown by the equivalent oxides given in Table 1, Martian regolith is rich in silica (SiO₂), constituting ~42%–47% of the regolith and ~49% of the crust—significantly higher than the 17%–25% typically found in Portland cement. Alumina (Al₂O₃) levels on Mars (~7%–10% in regolith, ~10.5% in crust) are also comparable to cement, where it generally accounts for <6%. Iron oxides—FeO (10%–26%) and Fe₂O₃ (4%–7%)—are present in substantial amounts, with FeO comprising ~18% of the bulk elemental composition of the Martian crust. Magnesium oxide (MgO) is similarly abundant (6%–9% in regolith, ~9% in crust). However, calcium oxide (CaO), the principal component of Portland cement (~60%–67%), is present on Mars in much lower concentrations (~5.7%–6.9%). This significant shortfall in CaO implies that *in situ* production of a true Portland cement analog on Mars is unlikely without external supplementation. In the near term, CaO would likely need to be imported from Earth, limiting the feasibility of producing standard Portland cement using only Martian-derived materials. It should be noted that the values presented here are equivalent oxides, representative of bulk composition rather than mineralogic species present; the oxides mentioned here generally will not be found in their oxide state on Mars.

**Table 1:** Chemical composition of Martian crust and regolith compared to Portland cement.

	Weight (%)							
Compound	SiO_2_	Al_2_O_3_	FeO	Fe_2_O_3_	MgO	CaO	SO_3_	Reference
Portland cement	17–25	≤6	–	≤6	≤6	60–67	<3	ASTM C150 (2023)
Martian Crust	49.3	10.5	18.2	–	9.1	6.9	–	Taylor and McLennan (2008)
Martian regolith	42.1–46.7	7.3–10.1	10.4–26.2	4.3–7.3	6–9.3	5.7–6.7	4.9–7.4	Clark et al. (1982), Gellert et al. (2004), Rieder et al. (2004), Morris et al. (2006)

### 2.2 Martian regolith as a substrate for biomineralization pathways

Given the presence of Si, Al, Fe, Mg, and Ca in Martian regolith, this section explores the potential of leveraging microbial metabolic pathways to generate biominerals from the local Martian materials, aligning with ISRU strategies. Crystalline silica (SiO₂), particularly as quartz, is prevalent in Martian regolith but is highly stable and chemically inert. Unlike redox-active elements such as Fe or Mn, Si does not participate in biological electron transfer reactions (Konhauser, 2016). Moreover, SiO_2_ is not metabolically useful to most microorganisms, offering neither energy nor a carbon source. Its low solubility at neutral pH (~0.1–1 ppm) further limits its bioavailability (Konhauser, 2016), making it unsuitable for direct microbial mineralization. However, microbes can indirectly contribute to the formation of cementitious materials when Si^2−^ interacts with other cations. For example, its reaction with Ca^2+^ can produce calcium silicate hydrate (C–S–H), the primary binding phase in Portland cement (Konhauser, 2016). Similarly, under alkaline conditions, interactions between SiO₂ and Al^3+^, Fe^3+^, or Mg^2+^ can yield geopolymers or alkali-activated materials with cement-like properties.

Although microbes do not directly precipitate Al, microbial activity can facilitate its incorporation into secondary minerals through bioleaching and weathering processes (Gadd, 2007). For instance, under acidic conditions, alumina dissolves in the presence of protons, releasing Al ions (Al^3+^) and water (Al₂O₃ + 6H^+^ → 2Al^3+^ + 3H₂O). The released Al^3+^ ions can subsequently interact with dissolved silica (SiO₂) and hydroxyl ions (OH^−^) to form aluminosilicate hydroxide structures (Al^3+^ + SiO₂ + OH^−^ → Al–Si–OH), precursors to clay minerals, such as biogenic formation of kaolinite(Gadd, 2007; Provis and Deventer, 2009).

Fe oxides can participate in biomineralization through dissimilatory iron reduction (DIR), a microbial process in which bacteria like *Shewanella* reduce Fe(III) to Fe(II) under anoxic conditions, leading to the precipitation of Fe minerals like magnetite (Fe₃O₄) and siderite (FeCO₃) (Lovley, 1991). However, neither magnetite nor siderite, common Fe-bearing minerals on the Martian surface, exhibit cementitious properties; they lack hydraulic activity and do not form binding hydrates like calcium silicate hydrate (C–S–H) or calcium aluminate hydrate (C–A–H), which are critical in Portland cement systems. Consequently, their binding capacity is insufficient to generate a hardened matrix capable of stabilizing mineral aggregates such as the Martian regolith. Nonetheless, due to its high density and Fe content, magnetite may serve as a functional additive for radiation-shielding concretes, relevant to systems designed to protect from cosmic and solar radiation (Hassler et al., 2014). Regarding MgO, some alkaliphilic microorganisms (e.g., cyanobacteria or sulfate-reducers) promote MgCO₃-based biomineral precipitation through alkalinization or photosynthesis (Hassler et al., 2014). MgO, upon hydration (MgO + H₂O → Mg(OH)₂), releases Mg^2+^ ions that can subsequently react with carbonate ions to form magnesium carbonates, such as hydromagnesite, nesquehonite, or magnesite.

CaO, which has the potential to facilitate calcium carbonate (CaCO_3_) precipitation via the biocementation process, is perhaps the most promising pathway for biomineralization for Martian applications. Microbially Induced Calcium carbonate Precipitation (MICP) has been the most extensively studied biomineralization pathway on Earth over the past two decades, with applications explored in soil stabilization (Mujah et al., 2017; Sharaky et al., 2018; Omoregie et al., 2020; Yin et al., 2022), mitigation of wind-induced desertification (Zomorodian et al., 2019; Fattahi et al., 2020; Devrani et al., 2021; Dubey et al., 2021), and sustainable building construction (Castro-Alonso et al., 2019; Iqbal et al., 2021; Bagga et al., 2022; Khoshtinat, 2023a). CaCO₃ precipitation can occur via several microbial mechanisms, typically classified into six categories: ureolysis, amino acid ammonification, photosynthesis, denitrification, sulfate reduction, and methanogenesis-induced pathways (Dupraz and Visscher, 2005; DeJong et al., 2010; Van Paassen et al., 2010; Achal et al., 2015; Zhu and Dittrich, 2016; Castro-Alonso et al., 2019). For brevity, only some of these mechanisms are discussed in detail here. Among these pathways, the ureolytic pathway is one of the most extensively studied. In this process, urease-producing bacteria, notably *Sporosarcina pasteurii*, catalyse the hydrolysis of urea into ammonia and carbonate ions. The production of ammonia increases the pH of the environment, which, in the presence of calcium ions, leads to the precipitation of CaCO₃ (CO(NH₂)₂ + 2H₂O + Ca^2+^ → 2NH₄^+^ + CaCO₃↓)(Pakbaz et al., 2018; Hosseini et al., 2024a). The second most extensively studied biocementation pathway involves photosynthetic microorganisms. In this mechanism, organisms such as cyanobacteria facilitate CaCO₃ precipitation through their metabolic activity. By consuming CO₂ during photosynthesis, they reduce dissolved CO₂ concentrations, leading to an increase in local pH. This shift alters the carbonate equilibrium, favoring the formation of carbonate ions, which then react with calcium ions to precipitate CaCO₃ (CO₂ + H₂O + light + Ca^2+^ → 2H^+^ + CaCO₃)(Billi and McKay, 2011; Sharma et al., 2020; Reinhardt et al., 2023). Table 2 shows a selection of the latest intriguing studies on biocementation via ureolysis and photosynthesis.

**Table 2:** Advancements in exploiting biocementation ureolysis and photosynthesis.

Pathway	Microorganism	Advantages	Disadvantages	References
Ureolysis	*Sporosarcina pasteurii*	- Efficient urease activity - Widely studied for MICP - High CaCO₃ yield	- Requires urea - Produces ammonia - Not photosynthetic	Pakbaz et al. (2018), He et al. (2024), Hosseini et al. (2024a)
	Thraustochytrium striatum	- Grows in dark - Uses urea and acetate - Works under Martian regolith simulation	- Needs oxygen - Produces ammonia	Gleaton et al. (2019)
Photosynthesis	*Chlamydomonas reinhardtii*	- Oxygen-producing - CO₂-consuming	- Needs light and water - Sensitive to Martian conditions	Sharma et al. (2020)
	*Haematococcus pluvialis*	- Tolerates low temperatures - Produces protective astaxanthin	- Needs light - Slow growth	Ariyanti and Handayani (2011)
	Cyanobacteria	- Use CO₂ and release O₂ - Survive in Martian-like atmospheres - Can be 3D-printed into structures	- Require light - Slow mineralization rate	Billi and McKay (2011), Verseux et al. (2016), Keller et al. (2023), Reinhardt et al. (2023), Tarasashvili et al. (2023)

### 2.3 Environmental constraints

Although Mars is considered to be very similar to Earth due to its rocky surface and axial tilt (~25.2°), which induces seasonal cycles, it differs markedly in environmental conditions. Mars has lower gravity (1.62 m/s^2^, ~1/3 that of Earth), no global magnetic field, and a thin CO₂-dominated atmosphere (~95.3%) with a relatively low surface pressure of 610 Pa (NASA, 2021). These factors expose the surface to intense cosmic and solar radiation and contribute to low average surface temperatures (−63 °C). These conditions significantly impact the viability and metabolic activity of microorganisms, as well as the hydration and curing behavior of cementitious materials. Few astrobiological studies have directly examined the effects of Martian environmental parameters on microbial activity, and none has investigated microbes with biomineralization potential. Simulated Martian conditions, low pressure, CO₂-rich atmosphere, and freeze–thaw cycles have been shown to markedly impair bacterial viability (Foster et al., 1978). However, certain psychrotrophic microbes can exhibit modest proliferation when moisture and nutrients are present, with up to three times increase in viable cells over 7–14 days. For example, *Escherichia coli*, *Serratia liquefaciens* (Berry et al., 2010), and *Psychrobacter cryohalolentis* (Zaccaria et al., 2024) have demonstrated moderate to high growth under specific Martian stressors. Nonetheless, overall biomass remains constrained due to desiccation and ionizing radiation.

While lower gravity can reduce structural loads, it also negatively impacts construction processes by disrupting particle settling and water distribution, which leads to non-uniform mixing, poor hydration kinetics, and increased porosity and microcracking, ultimately compromising material strength. Crystallization process experiments aboard the International Space Station revealed that microgravity suppresses sedimentation and bleeding, resulting in more uniform hydration but also in a 20% increase in porosity and larger pore sizes compared to Earth-based conditions, leading to weaker, more heterogeneous microstructures (Moraes Neves et al., 2019). In the context of biomineralization, low gravity presents additional challenges by impairing microbial adhesion and biofilm formation, thereby affecting the uniformity and efficacy of microbial-induced cementation. Mars’ extreme temperature fluctuations, from −90 °C in winter to 26.6 °C in summer, pose a significant barrier to biomineralization by disrupting microbial enzymatic activity. Even slight temperature shifts (1–2 °C) can alter enzymatic reaction rates by 10%–20% (Berry et al., 2010; Khoshtinat, 2023b). Moreover, most mesophilic bacteria become inactive near 5 °C, restricting biomineralization to a limited window of midday hours during Martian summer.

Elevated ultraviolet and ionizing radiation levels on Mars present a major challenge to microbial viability. While some *Bacillus* strains, such as *B. pumilus* SAFR-032, exhibit high resistance to Martian-like UV radiation (Newcombe et al., 2005; Osman et al., 2008), others, like *B. subtilis*, show reduced survival under regolith-shielded ionizing radiation (Moeller et al., 2010). In contrast, extremophiles like the cyanobacterium *Chroococcidiopsis* display resistance to both UV and ionizing radiation, making them more suitable candidates for Mars-based applications (Billi et al., 2000). These microorganisms have demonstrated the capacity to survive for days under simulated Martian conditions such as temperatures around −27 °C, a low-pressure CO₂-dominated atmosphere (~0.8 kPa), and direct exposure to Martian regolith simulants (Billi et al., 2019b; Keller et al., 2023). Moreover, their exceptional desiccation resistance, as demonstrated by the survival of dried biofilms for 672 days in the vacuum of space during the EXPOSE-R2 mission aboard the International Space Station, followed by successful revival upon rehydration, further underscores their potential for extraterrestrial applications (Baqué et al., 2013b; Billi et al., 2019a, 2019b).

## 3 Can biomineralization be adapted for Martian construction?

This section presents the authors’ perspective on the feasibility of applying biomineralization for Martian construction. It evaluates which biomineralization pathway is deemed suitable for Martian conditions and how it can be integrated with robotic systems and ISRU strategies. Additionally, it explores the potential of its incorporation into automated systems to achieve scalable, self-sustaining construction using local regolith. Finally, it outlines future research directions to address existing knowledge gaps.

### 3.1 Promising biomineralization pathway

Among the biomineralization pathways considered for Martian construction, biocementation appears the most promising due to its capacity to generate robust, cementitious materials suitable for extraterrestrial environments. As mentioned in Section 2.2, ureolysis and photosynthesis show particular promise for Martian construction applications. Table 3 summarizes the key concepts related to Martian regolith as a medium for biocementation discussed in Section 2.2.

**Table 3:** Comparison of biomineralization pathways for Martian application.

Element/oxide	Available weight (%)^*^	Biomineralization pathway	Advantage	Drawback
SiO₂	49.3	Indirect via interaction with Ca^2+^, Al^3+^, Fe^3+^, or Mg^2+^	Abundant in Martian regolith Forms key cementitious phases (C–S–H, geopolymers)	Chemically stable and low solubility Cannot be directly metabolized by microbes
Al₂O₃	10.5	Weathering/bioleaching to release Al^3+^, forming biogenic clays	Can participate in geopolymer or clay formation	No direct microbial precipitation Requires acidic conditions Limited structural strength
FeO / Fe₂O₃	26.2	DIR	Enables Fe^3+^ reduction under anoxic conditions Magnetite is useful for radiation shielding	Not cementitious (no binding gels) Poor hydraulic activity Limited to additive role in construction
MgO	9.3	Alkaliphilic bacteria	Promots MgCO₃ with cement-like binding properties Useful in dry, alkaline environments	Requires hydration and carbonate availability Limited durability vs. Ca-based systems
CaO	6.9	Biocementation	Widely investigated pathway High binding strength Compatible with Martian ISRU	Requires control of environmental pH and carbonate availability

^*^The maximum weight percentage value between crust and regolith from Table 1.

In this context, co-culturing microorganisms with distinct biocementation pathways and extreme environmental condition resistance can offer synergistic advantages. The proposed system features a synthetic bioreactor containing a co-culture of photosynthetic cyanobacteria *Chroococcidiopsis* and ureolytic bacteria *Sporosarcina pasteurii*. Although photosynthetic biocementation is significantly slower than ureolysis, the demonstrated resilience of *Chroococcidiopsis* under Martian-like conditions supports sustained production of oxygen and EPS, creating a favorable microenvironment for *S. pasteurii* and enhancing calcium carbonate precipitation in extraterrestrial settings. In this scenario, the bacterial co-culture would be applied to basaltic Martian regolith. Astronaut urine would supply the key ions (Ca^2+^, K^+^), supporting microbial growth and urea source for ureolysis. As demonstrated, a nutrient-rich “Martian medium” can be created by leaching regolith with water and enriching it with urine, achieving nutrient levels comparable to BG-11 medium (Concas et al., 2023). Photosynthetic activity by *Chroococcidiopsis* consumes dissolved CO₂ and HCO₃^−^, raising the pH and secreting EPS, providing a suitable microenvironment as a nucleation site for *S. pasteurii* CaCO₃ precipitation.

However, certain environmental controls are essential to ensure successful biocementation, leading the process to better performance in controlled, laboratory-like conditions. To maintain water in a stable liquid phase, a pressurized enclosure approximating Earth’s atmospheric pressure is preferred. Such pressurization also supports the retention of oxygen produced by photosynthetic microorganisms, gradually shifting the reactor environment from anoxic to oxic conditions, an essential transition for the aerobic metabolism of *Sporosarcina pasteurii*. Although *Chroococcidiopsis* have demonstrated resistance to ultraviolet (UV) radiation, incorporating a transparent UV-blocking membrane that excludes UVA and UVB wavelengths, the pressurized enclosure envelope can improve environmental conditions for *S. pasteurii*. This protective layer complements the intrinsic UV resilience of *Chroococcidiopsis* and helps maintain microbial viability and metabolic functionality. The biocementation reactor would operate through cyclic wet–dry phases: moistening facilitates microbial proliferation and calcium carbonate precipitation via ureolysis, while drying enhances structural consolidation and mineralization of the formed biocement (Baqué et al., 2013a,b; Gat et al., 2014; Mosca et al., 2019; Xu et al., 2020).

### 3.2 Integration with ISRU

From the authors’ perspective, biocementation is considered a promising biomineralization pathway due to its strong potential for integration with ISRU strategies, both during early Mars colonization and in later phases, where its byproducts could support secondary resource cycles. Key parameters include water and calcium, with additional substrates varying by pathway (e.g., urea for ureolysis and CO₂ and light for photosynthesis). Calcium, abundant in Martian basalt (McSween et al., 2009), can be extracted via leaching processes developed for metal or oxygen production, where it may arise as a byproduct. In case of crewed mission, urea can be derived from human waste (Zuo et al., 2023), creating a closed-loop system that repurposes metabolic waste for construction. Biocementation, when automated and adapted for Martian conditions, could support Mars’ development by integrating with ISRU systems, yielding byproducts such as oxygen (via photosynthesis) and ammonia (via ureolysis). It can function both as a construction method and as auxiliary support for critical operations. CO₂, a key input, also supports oxygen production via solid oxide electrolysis (Hoffman et al., 2022) and plant growth in greenhouses (Fackrell et al., 2024). In long-term scenarios, ammonia may also serve as a nitrogen source for Martian agriculture (Wamelink et al., 2014; Fackrell et al., 2024).

Water, the most critical aspect (Cheng and Cord-Ruwisch, 2012), may be sourced from subsurface ice or hydrated minerals. For example, radar observations from the Mars Advanced Radar for Subsurface and Ionospheric Sounding (MARSIS) instrument suggest the presence of stratified subsurface structures within the Medusae Fossae Formation, consistent with ice-rich deposits buried beneath approximately 300–600 m of dry overburden (Watters et al., 2024). In addition, bright basal radar reflections detected near the Martian south pole have been interpreted as possible subglacial liquid water bodies at depths of ~1.5 km of overlying ice (Orosei et al., 2018). However, certain precautions must be considered, as most compositional data currently available have been obtained through robotic missions, and the Mars Sample Return program continues to experience delays (NASA Sets Path to Return Mars Samples, Seeks Innovative Designs - NASA, 2024). Therefore, the extent to which such ice or water is contaminated with perchlorates, highly oxidizing and potentially toxic salts that also act as strong desiccants threatening organic life (Hecht et al., 2009; Quinn et al., 2011), cannot be determined until physical samples are returned to Earth. Consequently, the potential necessity of purifying any water extracted from Martian resources must be carefully considered for every use, whether its human use, agricultural use, or for ISRU/construction.

Regarding energy consumption, unlike thermal or microwave-based sintering of regolith reliant on solar, stored electrical, or nuclear energy (Nething et al., 2020; Reinhardt et al., 2023), biocementation operates at low temperatures with low energy demands, making it suitable for Mars’ limited power systems. However, the energy demand for extraterrestrial construction depends on multiple process-specific factors, including the processing method, local resource utilization potential, and the type of instrumentation and equipment used. Since adapting biocementation for lunar construction is impractical due to extreme daytime surface temperatures of ~127 °C (Chen et al., 2024), which surpass the thermal tolerance of the microorganisms involved. Additionally, biocementation is a relatively recent concept for Martian application, there is a lack of data regarding biocementation energy consumption for extraterrestrial use. Given the limited availability of extraterrestrial construction data, only a first-order comparison of energy consumption can be attempted by assuming that the material payload delivered from Earth is constant (e.g., hardware mass, volume, storage requirements, etc.) across different construction strategies. According to available data, the production of 1 tonne of CaCO₃ by biocementation on Earth consumes approximately 29.3 MJ in total (0.029 MJ/kg) (Deng et al., 2021). In contrast, conventional thermal sintering is estimated to require about 1,372 MJ/tonne (Spedding et al., 2020), while cold microwave sintering, reported to consume ~6.66 times less energy than thermal sintering (Liu et al., 2024), requires approximately 206 MJ/tonne. Although these values should be interpreted cautiously, they suggest that biocementation has a substantial energy efficiency advantage, on the order of 1–2 magnitudes, over other proposed extraterrestrial construction techniques.

### 3.3 Automation and remote operability

On Mars, where payload delivery from Earth is costly, additive manufacturing (AM) using Martian regolith as feedstock can significantly lower mission costs and complexity. AM, especially 3D printing, enables precise, automated, and resource-efficient construction (Azami et al., 2024). Its modularity and scalability allow infrastructure to grow with mission needs. Remotely commanded design updates to robotic AM systems enable rapid iteration and enable damaged structures to be repaired by reprinting parts as needed, which is crucial given limited resupply opportunities on Mars. Biocementation-based AM offers flexibility in material properties and construction methods techniques (Nething et al., 2020; Reinhardt et al., 2023; Pu et al., 2024). The process can extrude a slurry of regolith and microbes (Pu et al., 2024), enabling complex, strong geometries.

Advanced nozzles and multi-axis robotic arms will facilitate varied printing orientations, supporting arches and domes that withstand pressurization and external loads like dust storms. Since biocementation begins immediately upon mixing of feedstocks, precise control of mixing and flow rates is essential to prevent nozzle clogging from premature solidification. A conceptual biocementation nozzle may use a multi-channel system, delivering bacterial solution, nutrient media, and regolith slurry through separate feed lines with controlled flow rates. The bacterial solution is introduced last, just before extrusion, minimizing residence time and reducing the risk of clogging from premature calcium carbonate precipitation. This modular design enables real-time adjustment of mix ratios, allowing tailored material properties for specific structural or environmental needs. For Martian use, the nozzle would include dust-sealing features to mitigate contamination and abrasion, along with thermal controls, such as localized heating or insulation, to maintain fluid viscosity and microbial viability. Flow dynamics must also be calibrated for Mars’ reduced gravity to ensure consistent mixing and deposition across various print orientations.

Automating biocementation on Mars requires integrating regolith treatment with robotic systems capable of precisely delivering bacteria and reagents for CaCO_3_ precipitation. Rovers, aerial drones/helicopters, or subsurface crawlers could inject microbial cultures and nutrient solutions directly into the regolith. These robots would feature onboard reservoirs, automated mixing units, and sensor suites to control dosage and monitor key environmental parameters, including moisture, pH, and ion concentrations that govern microbial activity and calcite formation (Yang et al., 2023). Advanced robotic systems for biocementation could employ biosensors to detect microbial byproducts and noninvasive tools such as ground-penetrating radar, cone penetrometers, or ultrasound to monitor biocement spread and buildup. Onboard navigation systems (including imaging, positioning, and inertial sensors) enable precise surface or subsurface movement along predefined treatment paths. Remote operation allows for enhanced process control, while AI or machine learning can analyze sensor data to autonomously adjust injection timing, spatial targeting, and dosing. Operators can intervene as needed to ensure safety and responsiveness. Predictive models simulating fluid flow, microbial growth (Veiskarami et al., 2023; Mahdy et al., 2024; Khoshtinat and Marano, 2025; Khoshtinat et al., 2025), and geochemical reactions can further support real-time visualization and treatment planning. However, as previously mentioned, biocementation-based construction and additive manufacturing will work better in controlled conditions. Therefore, authors recommend that additive manufacturing-based biocementation processes be conducted in laboratory settings using high-fidelity Martian regolith simulants under controlled environmental conditions. This approach allows for systematic optimization of process parameters and microbial performance, thereby ensuring that methodologies can be rapidly adapted once actual Martian samples become available and once construction efforts on Mars begin in earnest.

## 4 Concluding remarks, challenges, and knowledge gaps

Given the relatively early stage of research on biocementation for Martian construction, significant gaps remain to be addressed. Biocementation for Martian construction is fundamentally multidisciplinary, requiring integration of microbiology, geochemistry, materials science, robotics, and construction engineering. Yet, current research often lacks the cross-disciplinary coordination needed to address its inherent complexity. Even on Earth, managing the interdependent factors (e.g., nutrient availability, microbial behavior, precipitation kinetics, and mechanical performance) is challenging. Martian conditions further complicate this through low gravity, high radiation, perchlorate-rich regolith, and low atmospheric pressure. In the absence of a standardized, system-level methodological framework, studies remain fragmented and difficult to compare, limiting progress toward viable implementation.

Developing planetary surface technologies requires testing under conditions that closely replicate the target environment’s gravity, temperature, atmospheric chemistry, pressure, and radiation environment. For biocementation systems designed to interact with Martian regolith, using compositionally accurate Martian regolith simulants is essential during research and development, as inadequate analogs compromise experimental validity, repeatability, and reproducibility. Although several Martian regolith simulants exist (Peters et al., 2008; Cannon et al., 2019; Long-Fox and Britt, 2023), Mars’ geological diversity further demands site-specific matching of particle size and geochemistry and large-scale testing in bulk simulant testbeds increases cost and complexity. Simulating Martian surface conditions (e.g., in thermal vacuum chambers with integrated radiation sources) remains technically challenging and resource-limited. Additionally, the effects of Martian gravity on microbial growth and biofilm dynamics are virtually unexplored, as current methods (e.g., parabolic and suborbital flights) offer only short-duration reduced gravity conditions and are hence unsuitable for sustained biocementation studies.

Fundamental biological questions remain unanswered, particularly regarding the response of bacteria to Martian conditions. While *Chroococcidiopsis* shows tolerance to desiccation and radiation its behavior in the presence of *S. pasteurii* under Martian stressors remains speculative. Gene expression under such conditions is largely uncharacterized, and the combined effects of these stressors on metabolic pathways, biocementation, and stress responses are unknown. Genomic/transcriptomic analyses under simulated Martian environments are essential but currently lacking. Without empirical insight into these organisms’ adaptation or failure under relevant Martian conditions, attempts at co-culture engineering remain premature. Dual-species systems may offer synergistic benefits, but microbial interactions are highly sensitive to environmental stress. Mutualism, competition, and allelopathy must be managed carefully in closed-loop systems where resource cycling and ecological stability are critical. Scarcity of nitrogen and phosphorus on Mars adds further complexity, requiring external supplementation or engineered metabolic pathways. To date, there is no empirical evidence supporting long-term co-culture stability under Martian constraints (Mosca et al., 2019; Billi et al., 2021; Koehle et al., 2023; Di Stefano et al., 2025).

Translating laboratory findings into functional technologies introduces further challenges. Bioreactor systems must withstand extreme accelerations during takeoff and landing, thermal cycling, handle abrasive regolith, and prevent clogging from CaCO_3_ precipitation and biofilm detachment. Gas exchange, water management, and light delivery must be optimized under Martian gravity and atmospheric conditions. Reactor scalability, robustness, and mass efficiency are nontrivial engineering hurdles, particularly when constrained by launch payload limits and ISRU strategies. Integration into life support systems for recycling waste gases, supplying oxygen, or generating construction materials requires comprehensive safety and reliability assessments. Without integrated, long-duration testing in analog or space environments, the pathway from concept to application remains highly speculative.

## Data Availability

The original contributions presented in the study are included in the article/supplementary material, further inquiries can be directed to the corresponding author.
